# Promoting risk reduction: increasing public health capacity to address dementia

**DOI:** 10.1093/geront/gnaf227

**Published:** 2025-10-01

**Authors:** Shelby Sutton Roberts, Mickal Lewis, Catherine Woodall Colcombe, Chelsea Kline, Jeanine Menczywor Donnelly, Benjamin Denno, Heather M Snyder, Matthew Baumgart

**Affiliations:** Division of Programs, Alzheimer’s Association, Chicago, Illinois, United States; Building Our Largest Dementia Infrastructure (BOLD) Public Health Center of Excellence on Dementia Risk Reduction, Alzheimer’s Association, Chicago, Illinois, United States; Division of Programs, Alzheimer’s Association, Chicago, Illinois, United States; Building Our Largest Dementia Infrastructure (BOLD) Public Health Center of Excellence on Dementia Risk Reduction, Alzheimer’s Association, Chicago, Illinois, United States; Division of Programs, Alzheimer’s Association, Chicago, Illinois, United States; Building Our Largest Dementia Infrastructure (BOLD) Public Health Center of Excellence on Dementia Risk Reduction, Alzheimer’s Association, Chicago, Illinois, United States; Division of Programs, Alzheimer’s Association, Chicago, Illinois, United States; Division of Programs, Alzheimer’s Association, Chicago, Illinois, United States; Division of Programs, Alzheimer’s Association, Chicago, Illinois, United States; Building Our Largest Dementia Infrastructure (BOLD) Public Health Center of Excellence on Dementia Risk Reduction, Alzheimer’s Association, Chicago, Illinois, United States; Division of Medical & Scientific Relations, Alzheimer’s Association, Chicago, Illinois, United States; Division of Programs, Alzheimer’s Association, Chicago, Illinois, United States

**Keywords:** Modifiable risk factors, Social determinants of health, Public health workforce, Risk reduction

## Abstract

The Alzheimer’s Association Building Our Largest Dementia (BOLD) Public Health Center of Excellence on Dementia Risk Reduction (Center), established in 2020, was created to assist public health agencies translate this science into action and build health department capacity to address dementia risk reduction. Through partnerships with the academic community and public health agencies, the Center provides tools, training, and data to support the integration of dementia risk reduction into existing chronic disease prevention and healthy aging efforts. This article outlines the Center’s approach, highlights key initiatives and outcomes, and identifies opportunities for future research to strengthen public health infrastructure to reduce dementia risk across the lifespan. Despite the strong evidence, dementia risk reduction remains underutilized in public health practice. By equipping the workforce with accessible training and resources and fostering collaboration across sectors, the Center creates a bi-directional bridge between research, public health practice and implementation.

## Promoting risk reduction: increasing public health capacity to address dementia

Public health works across the prevention spectrum to reduce risk for disease, increase awareness and access to early detection, and mitigate the negative effects of health conditions by supporting people living with disease and their caregivers (Alzheimer’s Association, n.d.). The role of public health in Alzheimer’s and dementia is no different. More than 7 million Americans aged 65 and older are living with Alzheimer’s dementia. This number is projected to rise to 13.8 million in 2060 (Alzheimer’s Association, 2025). Research now shows that certain health conditions and changeable behaviors—known collectively as “modifiable risk factors”—influence both the risk of cognitive decline and dementia (Baumgart et al., 2015; Livingston et al., 2024; Stephan et al., 2024; U.S. Department of Health and Human Services, 2024; World Health Organization, 2019).

In 2023, 69.5% of Americans had at least one of five modifiable risk factors for cognitive decline (midlife hypertension, midlife obesity, smoking, diabetes, and physical inactivity), and 34.2% had at least two of them (Centers for Disease Control and Prevention, 2024). The Lancet Dementia Commission estimated that as many as 45% of dementia cases worldwide could be attributed to modifiable risk factors (Livingston et al., 2024). Some studies have shown that the incidence and prevalence rates of dementia have declined over the past decades, particularly in high-income countries such as the United States and parts of Europe. Researchers have attributed this decline, at least in part, to a population with greater levels of education and better management of cardiovascular health, which likely impacts their dementia risk factor profiles (Satizabal et al., 2016; Wolters et al., 2020). However, these declines have not been experienced equally across all populations. Evidence shows that racial and ethnic disparities persist in dementia incidence, prevalence and outcomes (Mayeda et al., 2016).

A wide range of potential modifiable risk factors have been the subject of research studies—one review identified published journal articles on 364 different possible risk factors (Vitali et al., 2021)—with the Lancet Dementia Commission identifying 14 factors as having the strongest body of evidence less education, traumatic brain injury, hypertension, physical inactivity, diabetes, smoking, obesity, social isolation, air pollution, depression, excessive alcohol consumption, hearing loss, vision impairment, and hyperlipidemia (Livingston et al., 2024). As our understanding of the science of modifiable risk for Alzheimer’s and dementia continues to grow, the U.S. Department of Health and Human Services in 2021 added a sixth goal to the National Plan to Address Alzheimer’s Disease, the federal government’s strategic plan on dementia, to focus on reducing risk factors for dementia (U.S. Department of Health and Human Services, 2024). Scientific evidence of the impact of these modifiable risk factors on dementia risk creates opportunities for public health action to implement effective population-level intervention strategies, including by embedding health equity to address disparities.

Given the many modifiable risk factors and the close interrelation of some (e.g., obesity and physical inactivity), addressing multiple risk factors, including through multi-component interventions, is likely to be an effective strategy for reducing population-level dementia risk in the future. Interventions aimed at addressing and preventing particularly prevalent risk factors, such as midlife (age range of 45–65 years) hypertension, midlife obesity, and physical inactivity—each of which affects more than one in three adults—are poised to have significant public health impacts by improving physical and cardiovascular health and reducing dementia risk (Stephan et al., 2024).

The U.S. Congress recognized the need for greater public health capacity in addressing dementia by passing the Building Our Largest Dementia (BOLD) Infrastructure for Alzheimer’s Act (BOLD Act) in 2018. The BOLD Act authorizes program awards to state and local health departments, the creation of three Centers of Excellence, and additional support to the Centers for Disease Control and Prevention (CDC) to build a national public health infrastructure to promote dementia risk reduction, improve early detection and diagnosis of dementia, increase data analysis and timely reporting, and support dementia caregivers (United States, 2019). In 2020, as part of the implementation of the BOLD Act, the CDC named the Alzheimer’s Association as one of three national Public Health Centers of Excellence, each of which addresses a critical issue in public health’s role on dementia. Currently, 43 state, local, territorial, and tribal health organizations receive BOLD funding through the CDC to support their own public health efforts. The BOLD Act implementation efforts are driven nationally by the Healthy Brain Initiative Road Map (HBI Road Map) Series. The Road Map Series provides strategic directions to address dementia from a public health perspective by offering specific outcomes and actions health departments can take to support risk reduction efforts in their communities.

The Alzheimer’s Association BOLD Public Health Center of Excellence on Dementia Risk Reduction (Center) is a resource for state, local, territorial, and tribal public health agencies nationwide, including those with and without a BOLD program. The Center closely aligns with the outcomes and actions of the HBI Road Map Series. It has a crucial role in supporting key aspects of this framework and analyzing and synthesizing the research on dementia risk.

## Design and activities of the BOLD public health center of excellence on dementia risk reduction

The Center has three primary functions, as displayed in Table 1. To develop a Center of Excellence capable of achieving these functions, the Alzheimer’s Association designed a strategy to analyze the science around dementia risk, engage public health departments, and facilitate implementation and action. The resulting Center is led by the Alzheimer’s Association Care and Support Division, with support from the Alzheimer’s Association Division of Medical & Scientific Relations and partnerships with Wake Forest University School of Medicine (Wake Forest), the Association of State and Territorial Health Officials, the National Association of County and City Health Officials, Bridge Builder Strategies and Emory University Center for Public Health Training and Technical Assistance (Emory Centers). This collaborative group is structured to ensure expertise in the scientific evidence of dementia risk, in comprehensive program evaluation to demonstrate impact, and ability to reach to state and local public health departments.

**Table 1.** Primary functions of the BOLD public health center of excellence on dementia risk reduction.

Function	Description	Example actions
**Review scientific evidence**	Analyze the published evidence on risk factors for cognitive decline and dementia.	Developed science summaries for the modifiable risk factors and SDOH related to dementia risk.
**Translate evidence**	Create accessible information and resources for public health professionals.	Hosted the first Dementia Risk Reduction Summit and developed short videos on risk factors and a webinar series on SDOH.
**Build workforce capacity**	Equip the public health workforce with the ability to implement effective public health interventions.	Launched Community Convenings, Risk Reduction Learning Collaboratives, and the Public Health ECHO program to build state and local health department capacity.

*Note*. This table outlines the primary functions of the BOLD Public Health Center of Excellence on Dementia Risk Reduction, including reviewing scientific evidence, translating evidence into accessible information, and building workforce capacity to implement effective interventions.

BOLD = Building Our Largest Dementia; CME = continuing medical education; ECHO = extension for community healthcare outcomes; EPHS = 10 essential public health services; SDOH = social determinants of health.

To assess which risk factors have the greatest opportunity for public health action, the Center collaborated with Wake Forest to review the current body of scientific evidence through a public health lens. In 2021, Wake Forest convened a panel—comprising national and international scientists and researchers from disciplines including public health, epidemiology, social sciences, clinical practice, and aging research—to review, evaluate, and synthesize the current evidence. The panel initially identified 12 areas of possible focus and assessed their potential for significant public health impact. From these, the panel selected eight areas for the Center to focus on, based on the level of research support and strength of evidence for public health action. The eight areas were diabetes and obesity, physical activity, social engagement, diet and nutrition, vascular health, sleep, smoking and alcohol, and sensory impairments.

The eight areas highlighted the interconnectedness of potential modifiable risk factors, suggesting that a multifaceted approach to addressing risk for cognitive impairment and dementia may be an effective strategy across diverse populations. From this analysis, the Center developed scientific summaries for public health professionals to use and to guide the Center’s subsequent translation and implementation work. In 2022, the Center adopted a similar approach in analyzing the research on dementia risk related to the social determinants of health (SDOH). Wake Forest led a review, alongside a group of dementia and SDOH expert researchers, of the scientific evidence regarding SDOH and dementia risk. This review included a workshop at the 2022 Alzheimer’s Association International Conference^®^ (AAIC^®^). Based on this review, the following SDOH were identified as important for public health action due to their level of research support and strength of evidence: education, economics, food insecurity, racism, discrimination, inequity, and environment. Corresponding SDOH science summaries were developed and published on the Center’s website along with the risk factor summaries.

During its development and concurrently with the review of the risk factors, the Center established a leading states advisory group (LSAG), composed of health department leaders from Missouri, Tennessee and Washington State. This group advises the Center on the feasibility and usefulness of Center initiatives from the perspective of the health department. Multiple public health roundtables were also held to seek expert guidance in the development of resources from health departments beyond those represented in the LSAG. The recommendations from these gatherings informed the structure of the science summaries, the creation of resources for use in writing state Alzheimer’s plans, and the development of a SDOH toolkit and webinar series.

Building on this foundational work, the Center hosted the first national Dementia Risk Reduction Summit in May 2023. The event featured 35 experts to explore strategies for translating the science on risk factors and SDOH into public health action across the prevention intervention spectrum (Prevention Institute, n.d.). Nearly 400 people attended the Summit either in person or through the virtual livestream, including almost 100 state, local, territorial, and tribal public health officials who attended in person.

**Figure 1.**
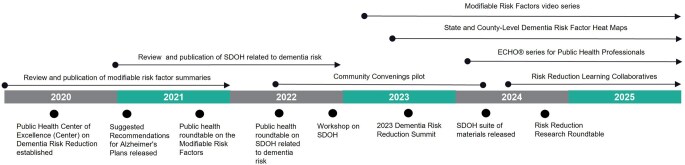
Timeline of the Public Health Center of Excellence on Dementia Risk Reduction’s national public health activities supporting dementia risk reduction from 2020 to 2025. ECHO = extension for community healthcare outcomes; SDOH = social determinants of health.

The Summit marked the halfway point of the Center’s 5-year cooperative agreement. The second half of the Center’s work has been focused on strengthening the capacity within state and local health departments to implement and integrate dementia risk reduction into their work. The Center is achieving this integration through various programs at the state and local level, hosting national webinars and in-person convenings, and continuing the development of implementation resources based on the initial scientific analysis. These efforts are described in Table 2 and discussed below in relation to the framework and outcomes of the Road Map. The Center also partners with Emory Centers to measure the effectiveness and impact of the Center’s work. Results presented in this manuscript, specifically in Table 2, are from the program evaluation conducted by Emory Centers. Through this design, the Center has been able to achieve its core functions, as described in Figure 1.

**Table 2.** Summary of dementia risk reduction initiatives and outcomes.

Project & brief summary	Reach	Outcomes	National availability	**EPHS & road map** **domain addressed**
**Community Convenings: A project where the Center assists local health departments in convening community leaders through an in-person, two-day workshop to create a dementia risk reduction action plan.**	185 attendees have participated in six Community Convenings; participating health departments include Alameda County, CA; Greendale, WI; Moore County, TN; City of South Tucson, AZ; Knox County, TN; and Nassau County, NY.	Across the six Community Convenings, 78.3% of attendees increased knowledge about SDOH related to dementia risk; 89.1% increased their understanding of how modifiable risk factors contribute to dementia; 84.8% developed strategies to address one or more modifiable risk factors in their communities; 97.8% felt motivated to implement their community action plan on dementia risk reduction.	The Community Convenings for Dementia Risk Reduction toolkit—guiding local health departments on how they can conduct a community convening in their jurisdiction—*Hosting a Community Convening* *for Dementia Risk Reduction: A Toolkit for Public Health Agencies* is now available on the Alzheimer’s Association Risk Reduction website.	P, M, W, E
**Risk Reduction Learning Collaboratives: An in-person, two-day workshop opportunity for local health departments to learn how to host a community convening on dementia risk reduction.**	64 health departments applied across 38 states and the District of Columbia. Efforts were taken to further recruit local health departments in the 16 states that did not have an applicant to participate in RLCs throughout the country from September 2024 to July 2025.	From the first six RLCs, 95.2% of evaluation respondents reported an increase in knowledge, and 93% reported an increase in confidence, including understanding the modifiable risk factors and SDOH, integrating risk reduction into existing work, hosting community convenings, implementing planned actions, and sustaining brain health efforts.	The seven RLCs were held in regions across the country, including the Southeast (Atlanta, GA), Southwest (Phoenix, AZ), South Central (Houston, TX), Midwest (Chicago, IL and Minnetonka, MN), Northeast (Boston, MA), and West Coast (Anaheim, CA).	P, M, W, E
**Dementia Risk Reduction Summit: A two-day gathering of national experts, leaders, and public health officials to explore ways public health can address risk factors for dementia.**	The Summit brought together 380 attendees: 230 in-person and 150 virtual. Approximately 100 in-person attendees were state, local, territorial, and tribal public health officials. The recording of the Summit has been viewed more than 2,400 times, significantly expanding its reach.	Among in-person attendees, 90% of evaluation respondents reported that the Summit provided valuable information applicable to their organization’s approach to dementia risk; 46% indicated that they had taken action within two months of the Summit, while 44% said they planned to take action in the near future.	Recordings of the Summit are available on the Alzheimer’s Association YouTube channel.	P, M, W, E
**Modifiable Risk Factor Summaries and Videos: 1–2-page written summaries and 10–15-minute videos that describe the science behind a variety of modifiable risk factors.**	As of June 2025: more than 1,500 cumulative downloads of the modifiable risk factor state-of-the-science summaries; more than 8,400 views of the modifiable risk factor videos; 1,515 people have enrolled to receive CME credits.		Summaries are available on the Alzheimer’s Association Public Health website. Videos are available on the Alzheimer’s Association YouTube channel.	W
**Social Determinants of Health Webinar Series, Infographics, Evidence One-Pagers, and Tool Kit: Suite of materials that describe the relationship between SDOH and dementia risk.**	5,840 cumulative registrants for the five-part webinar series; 2,100 cumulative live attendees with 2,580 views of the recording. As of June 2025, the SDOH evidence one-pagers have been downloaded 258 times. Similarly, the SDOH infographics have been downloaded 639 times.	Across the SDOH webinar series, 93.4% of attendees reported they gained knowledge of economic stability, nutrition and food security, social isolation, and healthy environments as they related to dementia risk; 93.1% found the webinars valuable to their work; 81.7% planned to apply the information learned through the series to enhance their public health efforts; 93.4% expressed interest in participating in similar webinars in the future.	Recordings of the webinar series are available on the Alzheimer’s Association YouTube channel. Infographics and evidence one-pagers are available on the Alzheimer’s Association Public Health website in addition to the newly published SDOH toolkit to assist public health in addressing SDOH in their communities.	W, E
**Alzheimer’s and Dementia Care ECHO^®^ Program for Public Health Professionals: ECHO^®^ six-session virtual education and training series for public health professionals on dementia risk reduction and how to integrate risk reduction activities into health department actions.**	Piloted by 34 local health departments, with 101 local public health officials in Tennessee participating.	Among ECHO^®^ session attendees, 95% participated in 4–6 sessions; 84% of evaluation respondents reported the series was an effective way to learn how to implement dementia risk reduction activities, increased their confidence in implementing these activities, and enhanced their overall job satisfaction.	The Center piloted two additional ECHO^®^ series in Washington state and Oklahoma in spring 2025. After the pilot is completed, the program will be available nationally for health departments through their local ECHO^®^ hubs.	P, M, W, E

*Note*. This table summarizes various dementia risk reduction initiatives, their reach, outcomes, and national availability data are from evaluations of the projects of the Center.

CME = continuing medical education; E = engage and educate the public domain; ECHO = extension for community healthcare outcomes; EPHS = 10 essential public health services; M = measure; evaluate, and utilize data domain; P = strengthen partnerships and policies domain; RLCs = risk reduction learning collaboratives; SDOH = social determinants of health; W = measure, evaluate, and utilize data domain.

## Impact of the public health center of excellence on dementia risk reduction on healthy brain initiative road map outcomes

The *Healthy Brain Initiative State and Local Road Map for Public Health, 2023–2027*, is a strategic guide designed to help state, local, and territorial public health practitioners address cognitive decline, dementia, early detection, risk reduction and caregiving in their communities (Alzheimer’s Association & Centers for Disease Control and Prevention, 2023). The Road Map outlines four key domains—Strengthening Partnerships and Policies, Measuring and Utilizing Data, Building a Diverse and Skilled Workforce, and Engaging and Educating the Public—each with specific outcomes and actions aimed at improving public health responses to cognitive health issues. The work of the Center in relation to the outcomes of the Road Map is described below and summarized in Table 1.

### Strengthen partnerships and policies

As discussed above, the risk factors for dementia span many existing public health strategies to reduce the risk for chronic disease. The role of public health departments as convenors and public health strategists offers the opportunity to increase community partnerships beyond the traditional partners in the dementia field (Centers for Disease Control and Prevention, 2017). The Center promotes a life course approach to dementia risk reduction, encouraging public health agencies to integrate partners who work on the risk factors across different populations and phases of life. One initiative of the Center, the Community Convenings program, fosters collaboration across local communities to address dementia risk factors. Through structured meetings, the program engages diverse community partners who focus on the risk factors for dementia most affecting their community. As a result of the convenings, participants have increased knowledge, capacity, and confidence in developing dementia risk reduction initiatives, action plans to address dementia risk factors and new partnerships with health systems, community organizations, faith-based groups, and schools, with the evaluation data described in Table 2. To increase the sustainability of action items developed in the Community Convenings, the Center trained health departments on how to integrate their goals and actions on dementia risk factors into existing programs and state or community health improvement plans. In fall 2024, the Community Convenings program expanded to the Risk Reduction Learning Collaboratives (RLCs). RLCs take a train-the-trainer approach by bringing together multiple local health departments to learn why and how to host a community convening on dementia risk reduction. The RLCs equip local health departments with the knowledge and resources necessary to build community partnerships, implement sustainable dementia risk reduction strategies and enhance public health initiatives as they prepare to host their own Community Convening (see Table 2 for program details, reach, and outcomes).

Through the Community Convenings and RLC programs, health departments have partnered with various community organizations, including libraries, community centers, universities, school systems, elected officials and other government agencies. Engaging these partners not only expands the reach of dementia risk reduction messaging, but the Community Convenings and RLC programs also introduce community leaders–many for the first time–to the connection between chronic diseases and dementia risk. This connection increases community awareness and strengthens the public health infrastructure by equipping local organizations with the knowledge and resources needed to effectively address dementia risk factors. These new partnerships also directly support the HBI Road Map outcome to increase integration with other chronic disease efforts by focusing on existing programs related to the risk factors for dementia and working to incorporate risk reduction messaging into those programs. Finally, to increase policy action and implementation, the Center developed a guide for state health departments, *Dementia Risk Reduction: Suggested Recommendations for Alzheimer’s Plans*, which provides specific examples of dementia risk reduction activities public health departments can integrate into State Alzheimer’s Disease Plans (Alzheimer’s Association, 2023). Increasing knowledge and awareness among leadership and policymakers is critical to ensuring the sustainability of these efforts.

### Measure, evaluate, and utilize data

**Figure 2.**
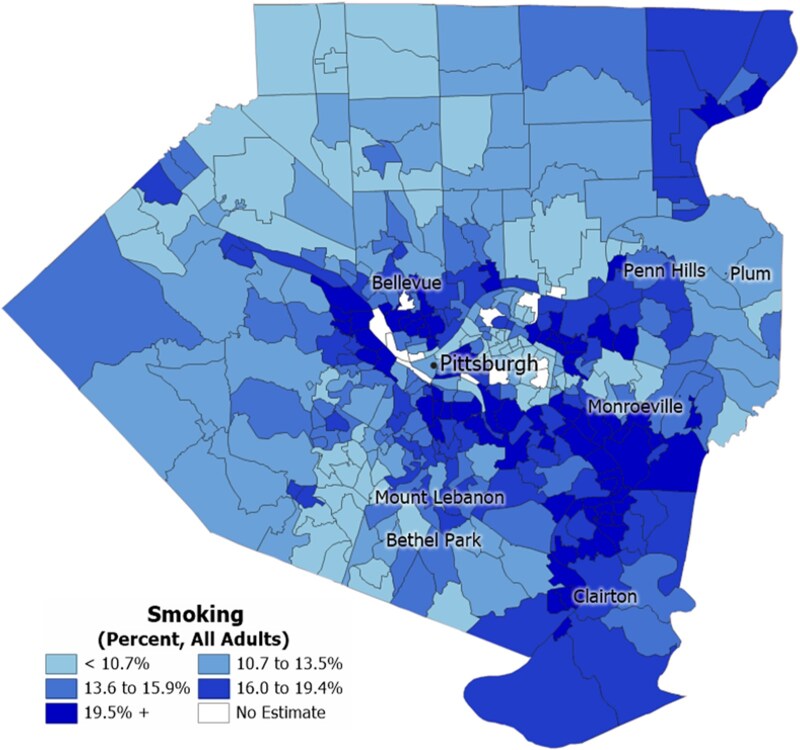
Example of a PLACES heat map showing geographic variation in dementia risk factor prevalence at the census tract level in Allegheny County, Pennsylvania.

**Figure 3.**
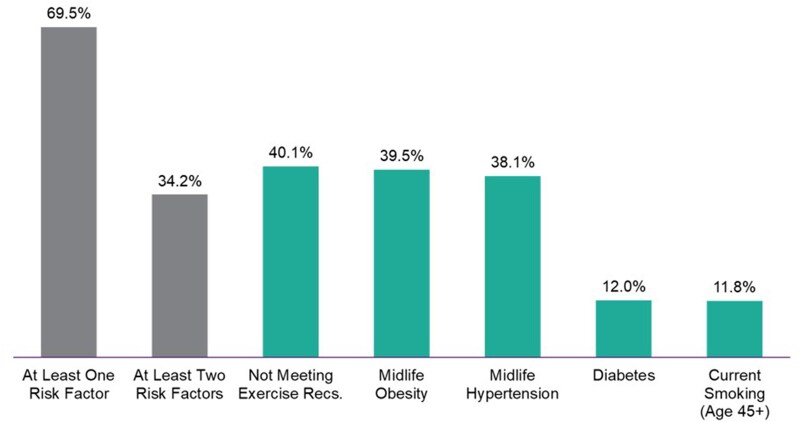
National prevalence of major risk factors for cognitive decline, based on 2024 BRFSS data. BRFSS = Behavioral Risk Factor Surveillance System; Recs. = Recommendations.

The Center uses data to inform decision-making and assesses community strengths and needs by helping public health departments identify the risk factors for cognitive decline and dementia most ripe for action in their states and communities. Using survey data from the Behavioral Risk Factor Surveillance System (BRFSS), the Center estimates the prevalence of six major risk factors for cognitive decline (mid-life hypertension, mid-life obesity, current smoking, diabetes, physical inactivity, and poor sleep) at the state level across various demographics, and provides ready-to-use fact sheets for health departments. Figure 3 provides national prevalence estimates for the six risk factors in 2023 and shows that 69.5% of the U.S. population has at least one of five major risk factors (not including poor sleep). The Center further supports local decision-making by creating risk factor prevalence heat maps at the county or city (by census-tract) or state (by county) level based on data from the Population Level Analysis and Community Estimates (PLACES) model (Centers for Disease Control and Prevention, 2024). Figure 2, an obesity rate heat map of Allegheny County, Pennsylvania, is one example of these custom visualizations of PLACES data and demonstrates that geographic variation in risk factor rates are evident even at local levels. This detailed view helps local decision-makers identify the areas with the greatest need for focused interventions and supports precise planning and resource allocation. To date, more than 1,750 heat maps have been created at the request of health departments.

### Build a diverse and skilled workforce

A skilled workforce representative of the community is key to the success of any public health initiative. Many of the Center’s activities involve creating a dementia-capable diverse public health workforce. The Extension for Community Healthcare Outcomes (ECHO^®^) series for Public Health Professionals is one successful model of building a diverse and skilled workforce. The program facilitates networking among local health departments, as health department staff participants increase their knowledge of and confidence discussing dementia risk reduction in their community. In 2024, using the framework of Project ECHO^®^ (Arora et al., 2007), the Alzheimer’s Association ECHO Superhub and the Center piloted the Alzheimer’s and Dementia Care ECHO^®^ Program for Public Health Professionals with 101 health department staff in Tennessee. This program aims to educate the public health workforce about modifiable risk factors for cognitive decline and dementia and how to integrate this knowledge into their chronic disease prevention strategies. Post-series surveys found that ECHO^®^ participants had, on average, a 47% higher self-rating on their knowledge about dementia risk reduction and a 27% higher self-rating on their confidence in implementing risk reduction strategies in their communities compared to before the course (see Table 2 for program details, reach, and outcomes). Building on this success, the ECHO^®^ Program for Public Health Professionals has since expanded in 2025 to Washington state and Oklahoma.

In addition to the ECHO series, the Center is building the workforce through webinars and online training. A webinar series featuring the SDOH and dementia risk had 5,236 registrants. Among the 1,800 cumulative live webinar attendees, 81.7% reported learning something they planned to implement in their work (see Table 2 for program details, reach, and outcomes). The Centers collaboration with the Alzheimer’s Association Healthy Brain Initiative (HBI) program, a funded recipient under CDC’s National Healthy Brain Initiative cooperative agreement (CDC-RFA-DP20-2003), led to the development of a dementia risk factor module to add to the *A Public Health Approach to Dementia*, interactive curriculum frequently used by public health schools and health departments to educate the current and future public health workforce. Increasing awareness of risk factors among public health professionals helps shift perceptions and conversations about dementia within communities, which can help achieve the goal of reducing stigma and bias about cognitive decline.

### Engage and enhance the public

An engaged and educated public is critical to advancing an understanding of dementia, the contributing risk factors and elevating reduction of those risk factors as a public health priority. Center materials have been used by medical professionals, community health workers, community education specialists, and others to work directly with individuals and communities with a goal of communicating about reducing dementia risk. Additionally, the Association used the Center’s analysis of the state of the science on modifiable risk factors to help develop *10 Healthy Habits for Your Brain*, a communications campaign designed to increase public knowledge about dementia risk reduction (Alzheimer’s Association, n.d.). These 10 concise, behavior-focused messages (protect your head, be smoke free, get moving, challenge your mind, control your blood pressure, manage diabetes, sleep well, stay in school, eat right, and maintain a healthy weight) give individuals specific actions they can take to reduce their risk of dementia. Launched in January 2024, the *10 Healthy Habits for Your Brain* campaign includes infographics and webpages as well as social media messaging (Alzheimer’s Association, n.d.). Materials are available in English, Spanish, Chinese, Korean, Vietnamese, Arabic, Bengali, and Haitian Creole, with additional translations under consideration. The Association’s chapter and volunteer networks have used the campaign as part of their community engagement. The resource complements the work of public health agencies in promoting risk reduction in the community and also provides an additional resource for health departments, who can share these simple risk reduction messages with individuals in their communities. The Center is able to provide printing and shipping assistance to health departments interested in using these materials in their community.

To further assist health departments in disseminating risk reduction messages, the Center is developing a communications toolkit based on national polling and focus groups that explored the public’s perceptions of dementia risk and their perceived ability to affect their own risk of cognitive decline and dementia. This toolkit will better empower health departments in increasing public awareness around dementia risk reduction.

## Implementation In practice: Tennessee’s dementia risk reduction efforts—a model for public health action

The Center’s work occurs through partnerships and action in health departments across the United States. One example of this partnership is the Center’s work with the Tennessee Department of Health (TDH). As part of the LSAG, TDH has provided critical expertise and guidance in the development of the Center’s dementia risk reduction materials and programs and has participated in and piloted many of these initiatives. The Center’s focus on reducing dementia risk through tailored tools and materials has aided TDH in more effectively addressing the needs of diverse communities, ensuring that public health strategies are both responsive and culturally appropriate. TDH has shared its progress and initiatives in national forums, such as the Dementia Risk Reduction Summit and the American Public Health Association annual meeting. Center and HBI webinars have also featured local health departments in Tennessee.

The TDH emboldened ECHO^®^ participants to integrate risk reduction strategies into existing public health initiatives and pursue Age-Friendly Public Health System (AFPHS) recognition, a framework designed to ensure public health systems meet the needs of older adults. Achieving AFPHS recognition promotes healthier aging, improves the quality of life for older adults, and supports communities in becoming more inclusive and supportive of their aging populations. Strong leadership from TDH has also resulted in increased action locally, with four local health departments participating in the Community Convenings and RLC programs. Through the Community Convenings program, Knox and Moore counties brought together local health systems, faith-based organizations, and other community partners to develop actionable plans for addressing dementia risk factors. These convenings facilitated conversations on the modifiable risk factors for dementia and SDOH related to dementia risk, helping embed risk reduction strategies into existing community health improvement efforts. The success of these efforts has increased community partnerships and the dementia-related knowledge and skills of the public health workforce within Tennessee. Obion and Weakley Counties participated in the RLC and are currently working on hosting their own local community convenings.

## Future directions and conclusion

The Alzheimer’s Association Public Health Center of Excellence on Dementia Risk Reduction was established to bridge the gap from research to practice, while also identifying new research questions that arise from practice. The Center’s goals are to advance the understanding, strengthen the evidence base, and support implementation strategies to reduce the risk of dementia across the general population. Over the past five years, the Center has directly engaged with the academic community through Roundtables and the first Summit on Dementia Risk Reduction as well as more than 210 health departments to build capacity for addressing dementia risk through Center’s initiatives, such as the ECHO^®^ Program for Public Health Professionals, Risk Reduction Learning Collaboratives, Public Health Roundtables and Community Convenings program. Hundreds of additional public health professionals have engaged in webinars and accessed the Center’s online resources. There has been a dramatic increase in requests from public health professionals for information and presentations on dementia risk reduction. At the same time, important questions continue to emerge for the academic community about evidence-based approaches to disseminate information and develop behavioral change programs aimed to reduce dementia risk. As the work progresses, the Center will continue to translate and actively share information, serve as a resource for health departments to share successes, and foster mutual learning through the expansion of learning collaboratives and national events. This collaboration will allow the public health field to continue to grow collectively in its capacity to address dementia risk.

As new insights into the strength and direction of the relationship between modifiable risk factors and risk for cognitive decline and dementia risk are discovered, the Center plays a key role in translating these complex findings into actionable tools, resources, and messaging for public health agencies and professionals. The Center also regularly updates statistics on the prevalence of risk factors as new data become available to help ensure public health professionals have access to the most up-to-date information for informed decision-making and effective community-based interventions.

The Center is committed to remaining a premier source for reliable public health information on dementia risk reduction through its partnerships with the academic community. It aims to support the growth of evidence-based interventions on reducing dementia risk and to provide health departments with the resources needed to implement risk-reduction strategies in their communities. Additionally, the Center will continue to offer opportunities for collaboration and discussion between academic institutions and health departments around dementia risk. The Center’s Roundtables offer bidirectional feedback to the research community highlighting gaps and areas where public health professionals need more evidence-base to continue to promote dementia risk reduction in their communities.

As public health efforts on dementia grow and expand, promoting risk reduction and encouraging the adoption of healthy habits across the lifespan must become a priority. This promotion will help reduce the risk of cognitive decline and possibly dementia and contribute to healthier communities. By collaborating with leading scientific experts and developing meaningful partnerships with health departments nationwide, the Center will continue to be a bi-directional bridge from research to public health practice to implementation, serving as a critical resource for the nation.

## Data Availability

This article does not report data and, therefore, the pre-registration and data availability requirements are not applicable.
